# Epigenetic Control of Infant B Cell Precursor Acute Lymphoblastic Leukemia

**DOI:** 10.3390/ijms22063127

**Published:** 2021-03-18

**Authors:** Oriol de Barrios, Maribel Parra

**Affiliations:** Lymphocyte Development and Disease Group, Josep Carreras Leukaemia Research Institute (IJC), Ctra. de Can Ruti, Camí de les Escoles s/n, 08916 Barcelona, Spain

**Keywords:** epigenetics, acute lymphoblastic leukemia, B lymphocytes

## Abstract

B-cell precursor acute lymphoblastic leukemia (BCP-ALL) is a highly aggressive malignancy, with poorer prognosis in infants than in adults. A genetic signature has been associated with this outcome but, remarkably, leukemogenesis is commonly triggered by genetic alterations of embryonic origin that involve the deregulation of chromatin remodelers. This review considers in depth how the alteration of epigenetic profiles (at DNA and histone levels) induces an aberrant phenotype in B lymphocyte progenitors by modulating the oncogenic drivers and tumor suppressors involved in key cancer hallmarks. DNA methylation patterns have been widely studied in BCP-ALL and their correlation with survival has been established. However, the effect of methylation on histone residues can be very different. For instance, methyltransferase *KMT2A* gene participates in chromosomal rearrangements with several partners, imposing an altered pattern of methylated H3K4 and H3K79 residues, enhancing oncogene promoter activation, and conferring a worse outcome on affected infants. In parallel, acetylation processes provide an additional layer of epigenetic regulation and can alter the chromatin conformation, enabling the binding of regulatory factors. Therefore, an integrated knowledge of all epigenetic disorders is essential to understand the molecular basis of BCP-ALL and to identify novel entry points that can be exploited to improve therapeutic options and disease prognosis.

## 1. Introduction

Acute lymphoblastic leukemia (ALL) derived from B lymphocytes has the highest annual incidence of all diagnosed childhood cancers in developed countries [1]. This type of leukemia can be subdivided into several subtypes, depending on the differentiation stage at which B lymphocytes acquire malignant properties that impair their proper development [2,3]. Recent research has allowed the implementation of patient stratification and more personalized therapies, which has improved the 5-year overall survival rate to as much as 90% [4]. However, the outcome of B-cell precursor ALL (BCP-ALL) in infants (younger than one year of age) is much more aggressive than in older children and adults, severely compromising their survival chances. In fact, a specific subgroup of patients harboring chromosomal rearrangements at the *KMT2A* gene (also known as *MLL*, mixed-lineage leukemia) is of special interest to basic researchers and clinicians, given its critically poor associated prognosis and elevated relapse frequency [5,6].

The gene expression profiling of infants diagnosed with BCP-ALL has revealed the existence of a genetic signature that determines the degree of aggressiveness and outcome of this malignancy [7,8]. Seminal work from Pieters’ laboratory in 2010 reported that the expression of the *HOX* homeobox gene family (*HOXA3*, *HOXA5*, *HOXA7*, *HOXA9,* and *HOXA10*) is correlated with a favorable prognosis [7]. *HOXA9* is frequently co-expressed with homeobox transcription factor *MEIS1* [9,10], and the combined downregulation of both genes prevents leukemic cells from proliferating uncontrollably [11]. In addition, the tyrosine kinase receptor FLT3 has become a widely used marker of prognosis and a potential therapeutic target [12,13,14], although FLT3 inhibitors have not yet succeeded in improving disease outcomes [15]. A recent genome-wide analysis of a large cohort of BCP-ALL infants has identified a novel genetic signature that can help predict disease progression and patient outcomes [8]. Therefore, patients with elevated expression of CDK inhibitors (such as p21 and p16), *TGFB1* or *SMAD* and reduced levels of genes involved in DNA integrity preservation and DNA repair (e.g., *CHEK1*, *ATM*, and *BRCA1*) are classified among the poor-prognosis subtypes of BCP-ALL [8].

The expression of these and other genes that act as essential drivers of BCP-ALL is driven by several mechanisms. The multistep process by which the immune system is built up during embryogenesis requires strict control during the B lymphocyte differentiation process [16]. However, this strict equilibrium can be deregulated by genetic alterations (e.g., fusion genes generated by chromosomal translocations or hyperdiploidy) that take place before birth, leading to an aberrant differentiation stage considered to be a pre-leukemic clone [3,6]. Although a variety of hypotheses have been proposed [3,17], it is believed that a prenatal secondary hit completes the onset of an overt BCP-ALL. Some genes involved in the initial genetic alterations (such as *KMT2A*) exert a key function in altering target gene expression by epigenetic remodeling and regulate hematopoietic system development in mammals [18,19]. In addition, most primary and secondary hits involve the deregulation of epigenetic mechanisms, either by directly modifying DNA or by specifically altering chromatin epigenetic marks. It must be noted that the aberrant behavior of these genes, involved in epigenetic regulation and chromatin remodeling, is more frequent in infant BCP-ALL than in adults, especially in recurrent cases [20] Here, we review the most relevant epigenetic mechanisms that aberrantly alter gene expression in BCP-ALL.

## 2. Epigenetic Alterations That Affect Gene Expression in BCP-ALL

### 2.1. Role of Epigenetic Modifications in Cancer

Epigenetics refers to the mechanisms that modify gene expression in a reversible way, without introducing permanent alterations in the DNA sequence itself [21]. However, an inappropriate expression of specific genes due epigenetic mutations can be inherited, even though the primary coding sequence remains unaltered [22]. The most common epigenetic marks consist of covalent modifications that can affect DNA nucleotides and histones [21]. These mechanisms are frequently found to be aberrantly deregulated in several types of cancer [22,23]. The commonest types of epigenetic alterations are DNA methylation in cytosine residues, which are associated with gene repression, and histone modifications, which mainly occur through the methylation, acetylation, or ubiquitination of specific residues that have distinct effects on gene expression depending on the residue and genomic region affected [21,22]. The ways by which epigenetic modifications influence progression of BCP-ALL are summarized in Table 1 and described in further detail in the following sections.

### 2.2. Histone Methylation Pattern Alterations

The genetic alterations that drive the onset of childhood leukemia usually involve genes that alter the methylation pattern of specific histones. The most common alterations are detailed below.

#### 2.2.1. Histone 3 Lysine 4 (H3K4) and Lysine 79 (H3K79) Methylation

The translocation of long arm (q) of chromosome 11 at distant genome locations usually constitutes an initial pre-leukemic hit in BCP-ALL, which occurs during embryonic development [24,72]. This translocation dramatically alters the pattern of histone methylation in the affected cells, since it involves the *KMT2A* gene (located in band 11q23), which harbors methyltransferase activity at the histone 3 lysine 4 (H3K4) residue with the capacity to transcriptionally activate gene targets during normal development, by increasing the levels of trimethylated H3K4 (H3K4me3) [24]. This is facilitated by a catalytic SET domain with an ancestral origin, which methylates lysine residues on histones. For this to function, KMT2A requires the cooperation of other cofactors, such as WDR5, RbBP5, ASH2L, and DPY-30 [25,26], to acquire methyltransferase activity. For instance, the absence of WDR5 prevents H3K4 from being methylated [73,74]. Consequently, the strategy of blocking KMT2A-WDR5 association is a promising approach in terms of KMT2A-translocated ALL treatment [75]. Other studies have ruled out the possibility that WDR5 could have a pivotal role, attributing instead a key function to the heterodimer RbBP5-ASH2L as the minimal unit required to conserve KMT2A catalytic activity [76].

The KMT2A protein can function as a transcriptional activator or repressor. This dual role depends on a specific region built up of homeodomains PHD1-4 and an adjacent bromodomain (BD). This structure determines KMT2A protein stability and confers plasticity to its transcriptional activity [77,78]. However, point mutations that disrupt the correct formation of this protein region can prevent KMT2A from binding to the BMI1 repressor complex, restricting its activity to gene induction. The outcome of BCP-ALL patients (including infants) with this alteration is poor compared with that of patients with an intact PHD1-4/BD structure [27].

Chromosomal rearrangement of KMT2A generates aberrant fusion proteins with other factors, leading to the modification of their transcriptional activity or resulting in alterations of its own mechanisms. Although more than 70 translocation partners for KMT2A have been reported, four of them (AF4, AF9, AF10, and ENL, located on chromosomes 4, 9, 10, and 19, respectively) account for more than 90% of infant ALL cases [27,28]. When the KMT2A gene fuses with any of these partners, its methyltransferase activity is affected, which leads to aberrantly altered chromatin states, resulting in a deregulated expression of specific genes that require strict regulation for the proper and controlled development of B lymphocytes [18,79].

The alteration of methyltransferase activity of KMT2A upon genome rearrangement combines with an enhanced ability to recruit chromatin remodelers, incorporated through its new fusion partners, such as AF4. Remarkably, AF4, AF9, AF10, and ENL partners belong to the super elongation complex (SEC) [35,36], together with other methyltransferases such as DOT1L, NSD1, and CARM1 [37]. These enzymes impose histone methylation marks that facilitate an open conformation of chromatin, thereby enhancing gene transcription. The main modifications introduced by SEC proteins are H3K36me2/3, H3K79me2/3, H3R17me2a, and H4K20me1 [37,38]. An aberrantly increased H3K79me3 mark is one of the main hallmarks of KMT2A-rearranged BCP-ALL, a process occurring through the altered activity of the DOT1L enzyme, which is recruited to proximal transcribed regions of active genes [28,80,81]. The increase of H3K79me2/3 levels caused by DOT1L involves the downregulation of monomethylated H3K79 residues [81] and a biased proportion of H3K4me3:H3K79me3 levels towards the latter [82]. This conversion is correlated with a dynamic change from low to high levels of gene transcription. In addition, DOT1L recruitment prompts the enrichment in H3K79me marks located at intergenic sequences, which act as enhancers that induce large distance-located promoter regions [81]. It is worth mentioning that the altered function of methylating enzymes like DOT1L or NSD2 in BCP-ALL does not lie on an altered protein expression, rather than in a differential binding affinity with their partners, as a result of genomic rearrangements that give birth to aberrant fusion proteins like KMT2A-AF4.

The general pattern of regions bound by KMT2A-AF4 in BCP-ALL cells is characterized by narrow H3K79me2/3 and H3K36me3 picks at gene promoter and enhancers. However, a small but significant proportion of genes show KMT2A-AF4 spreading through the gene body. This subset of genes includes some prognosis markers, like CPEB2, MBNL1, RUNX2, and ZEB2, and is characterized by elevated levels of H3K79me2/3 marks, to the detriment of H3K36me3, indicating greater DOT1L activity in these regions [39]. Among other activities, DOT1L represses SIRT1 histone deacetylase, which enriches chromatin with repressive marks, such as H3K9me3, thereby boosting the presence of active enhancer region marks, like H3K27ac [40,42]. Consequently, the methylation of H3K79 residue mediated by DOT1L, combined with SIRT1 inhibition, involves increased chromatin decoration with active, rather than repressive, marks [40]. This increased chromatin accessibility provides a great opportunity for the binding of transcription factors, thereby activating the enhancer function, and increasing the frequency of the interaction with the target promoter. ELF1 is one of the transcription factors whose binding frequency increases when the H3K79me2/3 mark is enriched [40].

In a positive feedback loop, the KMT2A-AF4 fusion protein activates a single member of the SEC, the P-TEFb kinase, which triggers the activity of promoter-arrested RNA polymerase II, and ultimately alters the pattern of the ALL transcriptional profile [38]. In parallel, the PAF1 complex, which is associated with RNA polymerase II, and monitors H3K4 and H3K79 methylation through the involvement of the ubiquitin ligase complex E2/E3 [27,29]. However, the presence of homeobox gene IRX1 impairs the ability of the KMT2A-AF4 fusion protein to bind endogenous cofactors, such as the SEC, and limits the potential recognition of H3K79me3 signatures. In consequence, IRX1 overexpression blocks the induction of leukemogenic drivers such as MEIS1 [83]. Intriguingly, IRX1 has an opposite oncogenic role, by maintaining a quiescent stem-cell state through the enhanced expression of HOXB4 and EGR family members, which increases the incidence of relapse in infants with high levels of IRX1 expression [83,84].

The hypermethylation of CpG islands (CGIs) at promoter regions is usually associated with inactive chromatin, whereas histone methylation may have an opposite effect. For instance, H3K4me3 and H3K79me2/3 are associated with unmethylated promoters, which facilitates gene transcription [41]. KMT2A-fusion proteins generally bind to regions of unmethylated CpG islands (uCpGs), and their recruitment to these areas is controlled by other cofactors such as Menin/LEDGF or PAF1 complex [30,31,32]. Studies from Thomas Milne’s laboratory have demonstrated a close coincidence between KMT2A-AF4 and Menin cofactor binding sites, rather than with PAF1 complex, in poor-prognosis BCP-ALL infants [39]. Given the difficulties of trying to directly target KMT2A-AF4, these studies point to Menin as a suitable cofactor for targeting with novel therapeutic strategies. In fact, several compounds (e.g., MI-2 and MI-538) with the ability to interfere with KMT2A-Menin binding have been developed, offering a promising strategy for preventing KMT2A binding to specific genomic sites and for activating its direct targets [85].

#### 2.2.2. Histone 3 Lysine 27 (H3K27) Methylation

Lysine 27 at histone 3 (H3K27) is an important location for epigenetic regulation. Its strict modulation at enhancer regions may determine the expression of distant promoters. Trimethylation of this specific residue induces a close chromatin conformation, as is very typical in lymphomas, and which is mediated by gain-of-function of Polycomb member EZH2 [50]. However, there is a converse H3K27me3 pattern in BCP-ALL, with decreased methylation and an increased proportion of unmodified or acetylated residues, since EZH2 usually displays loss-of-function mutations in this malignancy [45]. Given the relevance of the H3K27 mark, high-resolution chromosome conformation capture (Hi-C) data have provided insight into the mechanisms underlying gene expression in B lymphocytes [86]. An example of this is the regulation of *FLT3*, an oncogenic driver of BCP ALL [12]. Hi-C data revealed the activated expression of the *FLT3* gene in patients with a specific deletion in chromosome 13 (13q12.2). This deletion facilitates increased interactions between H3K4me3 marks at the *FLT3* promoter and the H3K27ac activation marks, which are located at an enhancer element within the gene body of the *PAN3* gene [33].

Likewise, the importance of H3K27ac marks in determining chromatin accessibility to enhancer regions can help predict how BCP-ALL patients will respond to standard established glucocorticoid therapy. The enrichment in H3K27ac marks within enhancer regions yields an open chromatin conformation and facilitates the recruitment of CTCF transcription factor in cooperation with the glucocorticoid receptor (GR), which induces target genes such as *BIM* [55]. Accordingly, the *BIM* enhancer region was found to be demethylated in ALL glucocorticoid-sensitive cells, partially through the recruitment of PU.1, which enables the increased acetylation of H3K27 residue [87]. As a consequence, infants with high levels of H3K27ac enhancer marks respond positively to dexamethasone treatment, whereas those with a low level of enrichment of this epigenetic mark are resistant to the therapy [55].

**Figure 1:**
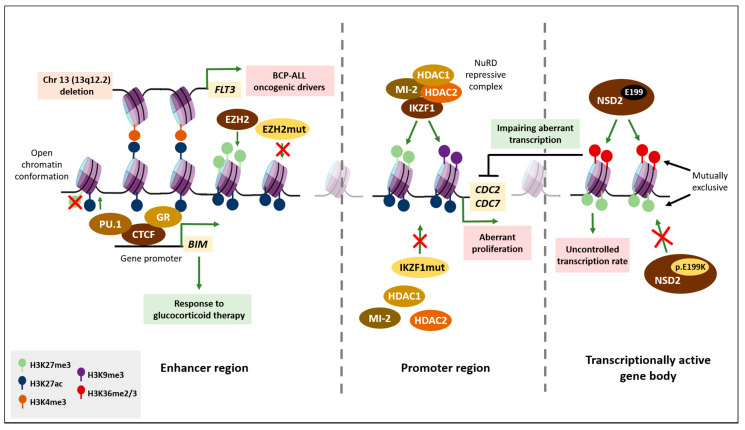
Dual effects of epigenetic regulation of H3K27 in BCP-ALL. Schematic picture of how epigenetic alterations of the H3K27 residue affect gene regulation and key biological processes that guide the progression of BCP-ALL. Green arrows indicate activation; red lines and crosses indicate repression. Black line indicates control of uncontrolled transcription rate. Green squares indicate biological processes that improve BCP-ALL outcome; red squares indicate processes that induce poorer prognosis. Distinct colors have been assigned to each histone modification mark, as indicated in the Figure Legend.

The importance of epigenetic regulation of the H3K27 residue at distinct regulatory regions of BCP-ALL regulators is shown in Figure 1.

#### 2.2.3. Histone 3 Lysine 9 (H3K9) and Lysine 36 (H3K36) Methylation

The methylation of H3K27 can also be regulated by the B cell development transcriptional regulator IKZF1 (also known as Ikaros). It is generally considered to be a tumor suppressor in BCP-ALL [51], given its reported capacity to enrich chromatin with H3K27me3 residues. This effect is mediated by the associated NuRD repressive complex, which includes HDAC1, HDAC2, and MI-2 [43,44]. In addition, the proportion of H3K9ac mark skews towards H3K9me3 in the presence of Ikaros and its associated factors, conferring a repressive state to chromatin. Therefore, Ikaros exerts an antioncogenic role, mediated through the repression of some targets involved in cell cycle progression (e.g., *CDC2* and *CDC7*), that counteracts *EZH2* loss-of-function in BCP-ALL [44]. Pediatric patients with a mutated or impaired Ikaros function are classified as high-risk individuals among BCP-ALL cases [88]. Nonetheless, the function of Ikaros in BCP-ALL merits further study, given that recent data demonstrate it can be induced by the KMT2A-AF4 fusion protein [34]. Consequently, it is tempting to speculate that the repressive role of Ikaros may somehow contribute to the aggressive phenotype presented by patients with t(4;11) rearrangement.

Another key regulatory residue in terms of leukemic cells transformation is lysine 36 in histone 3 (H3K36). In general, bodies of transcriptionally active genes are decorated with H3K36me2/3 marks, which impair aberrant transcriptional initiation within coding sequences [46]. However, H3K36 is the target of recurrent epigenetic modifications that drive some pediatric cancers. The H3K27me3 and H3K36me3 marks are mutually exclusive, and this antagonism determines the final methylation site and the degree of chromatin accessibility [47]. H3K36 residue methylation is catalyzed by NSD2 methyltransferase, whose catalytic activity depends on the E1099 residue [45,48,49]. A small but significant percentage of infant BCP-ALL cells present a point mutation in this glutamate residue (p.E1099K), which aberrantly triggers cell proliferation. The reversion or depletion of this mutation drastically blocks leukemogenic activity, which makes NSD2 targeting a promising therapy in hematological malignancies [45].

#### 2.2.4. Other Histone Alterations Affecting Methylation Patterns

The methylation of histone arginine residues can also influence gene expression, although it has not been thoroughly studied [52]. The regulation of arginine methylation is mediated by protein arginine methyltransferase 5 (PRMT5), which imposes symmetrical dimethylation in substrates like histones H4 and H2A in BCP-ALL [53]. Through its action on the H4R3 residue, PRMT5 inhibits transcriptional induction at the promoter site of its targets, which include genes, like *CLC* and *CTSB*, that are involved in proper B cell differentiation and apoptosis [52,54]. Recently, upregulation of symmetric dimethylation of H4R3 (H4R3sme2) has been described in newly diagnosed BCP-ALL infants, as well as in relapsed cases, whereas this mark levels are reduced during remission [52].

KMT2B (also known as MLL2) shares a similar structure (including the SET domain) and methyltransferase function with KMT2A protein [89]. Nevertheless, no translocation of *KMT2B* has been reported in BCP-ALL patients [90], whereas missense mutations and the aberrant activation of its expression have been linked to impaired survival in hematological malignancies [91]. In this sense, KMT2B has been proposed as a potential target in *KMT2A*-rearranged leukemia, given that the deletion of KMT2A-AF9-transformed cells reduces viability and proliferative capacity [92]. Conversely, KMT2B is involved in chromatin silencing via the recruitment of SIRT1. In this case, KMT2B would exert a tumor suppressor function [93].

### 2.3. DNA Methylation

Recent advances in high-throughput DNA sequencing to detect epigenetic changes have considerably increased our knowledge about the importance of epigenetics in leukemogenesis, contributing to the conception that DNA methylation can be used as a biomarker for BCP-ALL classification and outcome prediction [94]. Infant patients who suffer ALL usually experience an adverse outcome, influenced by the high relapse incidence of the disease. Therefore, good biomarkers of potential relapse are highly beneficial for identifying infants at risk of relapse after being treated with current therapies. DNA methylation status can be used both as a biomarker and as a therapy target since it can be reverted with demethylating drugs.

The availability of DNA methylomes from distinct types of cancer cells has revealed that DNA methylation is frequently altered in leukemic cells and that boundaries between methylated and unmethylated regions are often deregulated [95]. The excessive hypermethylation of CpG islands (CGIs) is the most widely studied of the various alterations affecting DNA methylation [96]. Several studies have demonstrated that BCP-ALL cells display a predominant hypermethylation pattern in CGIs, a general pattern that is shared by all genetic subtypes, commonly affecting the promoter regions of tumor suppressor genes. This pattern of methylation is more frequent in infants and children than in adults, despite the fact that some key gene promoters have been also found hypermethylated in adult B-ALL [97]. Several studies have reported higher methylation levels in malignant cells than in normal hematopoietic B-cell precursors and in matched remission samples of the same patients [98,99,100,101]. It can therefore be concluded that many epigenetic changes are required to produce malignant leukemic transformation. Most of the changes occur in regions largely occupied by H3K4me3 and H3K27me3 modifications (discussed in Section 2.2 of this review), which affect genes involved in the loss of plasticity and maintenance of self-renewal capacity [102].

Each ALL subtype (defined by its initiating hits, such as chromosomal translocations) harbors a set of differentially methylated CpGs. These signatures can be used to distinguish between several subtypes:

- High hyperdiploid (HeH) ALL: These cells have a stronger hypomethylation signature than other subtypes of this malignancy [103]. An approximately 5% lower level of genome methylated CpGs has been found in HeH ALL cells compared with normal B cell precursors [102].
- *ETV6*-*RUNX1* ALL: Cells from this ALL subtype tend to present high levels of DNA methylation [97]. However, the level of expression of *IGF2BP1*, *EPOR*, *FUCA1* and *HLA-DPB1* is higher due to hypomethylation of the promoter regions of these genes [94].
- *KMT2A*-rearranged ALL: These cases display a greater degree of DNA methylation, mainly in patients with t(4;11) and t(19;11) translocations, compared with normal bone marrow cells. Levels of methylation of t(9;11)-translocated patients much more closely resemble healthy bone marrow samples [41,94].
- *TCF3*-*PBX1*: Given the low incidence of this subtype, DNA methylation levels in these patients have not been widely studied. However, the signature seems mostly to be hypomethylated [104].
- *BCR-ABL1* ALL: The Philadelphia chromosome is an indicator of poor prognosis in ALL, and targeted therapies with imatinib or dasatinib are used to treat these patients [104]. Remarkably, DNA methylation plays a secondary role in these patients. In fact, only 271 subtype-specific differentially methylated CpG sites in 36 genes were detected in the *BCR-ABL1* ALL subtype, while other subtypes harbored around 2000 differentially methylated CpG sites [100]. The effect of DNA methylation in *BCR-ABL1* ALL is probably indirect, and the aberrant alterations in gene expression are far from being a direct consequence of DNA methylation pattern modifications [94].
- Intrachromosomal amplification of chromosome 21 (iAMP21): This subtype has not been widely studied because of its low incidence. However, the signatures of iAMP21 and HeH cases overlap to some extent [100].

One of the first studies to investigate DNA methylation in BCP-ALL considered chromosome 3, given the presence of several tumor suppressor genes located within its short arm [98]. The *PPP2R3A* gene was methylated in 82% of BCP-ALL cases, while *FBLN2* and *THRB* displayed methylation frequencies over 55%. These methylation patterns were specific to B lymphocyte-derived ALL, rather than to T-ALL [98]. In all cases, the excess methylation was accompanied by the silencing of gene expression.

Some years later, studies of small groups of genes revealed that patients with low levels of methylation are more prone to relapse. However, it has proved difficult to reproduce these results, since methylation signatures present at relapse are rare at the time of diagnosis, which makes it difficult to foresee potential relapses depending on the initial methylation signatures [100,105]. For instance, the *CDKN2A*, *PTPRO,* and *COL6A2* genes, which commonly behave as tumor suppressors, are hypermethylated during relapse [106]. Likewise, there is a clear overlap between the CGI methylation pattern in ALL relapse and that of genes regulated during embryonic development [100]. This finding reinforces the fact that BCP-ALL originates during embryonic development and that uncontrolled DNA methylation during this period can result in a poor-prognosis ALL, with a high probability of relapse after initial therapy.

Some genes with an essential role in hematopoietic diseases display an altered methylation pattern within their promoter regions in the context of ALL. These alterations strongly affect the leukemic cell phenotype and disease outcome [107]. This is the case for several tumor suppressor genes, like *CDKN2B* (also known as p15), *CDKN1C* (p57), *DLC1,* and the JAK-STAT-negative regulator *PTPN6*, whose promoters become aberrantly hypermethylated, causing their expression to be downregulated in BCP-ALL [108,109,110,111,112]. For *CDKN1C*, promoter hypermethylation is associated with an adverse outcome [113]. The tumor suppressor *TP73*, which has a similar function and structure to p53, is hypermethylated in 20-55% of childhood BCP-ALL cases [114], thereby blocking the apoptotic capacity of leukemic cells. Likewise, t(4;11) BCP-ALL patients have hypermethylated *DLX* gene family members (*DLX3* and *DLX4*), which also interferes with apoptotic programs [115]. Conversely, *SALL4* gene, which cooperates in maintaining self-renewal of stem cells, displays promoter hypomethylation in BCP-ALL [116].

No solid correlation with promoter DNA methylation has been observed for *IKZF1* and *TET2*, two important mediators of B-cell differentiation [107]. *IKZF1* promoter is not methylated in healthy B cells [117] and displays a hypermethylation pattern in solid tumors (e.g., lung cancer), although no alterations have been reported for BCP-ALL patients. Similarly, *TET2* promoter methylation is not affected in infant ALL [118], whereas it is clearly hypermethylated in acute myeloid leukemia (AML) patients [119].

As discussed in Section 2.2, the *KMT2A* gene can undergo several rearrangements with alternative partners that confer distinct degrees of aggressiveness on the resulting leukemic clones. Remarkably, a cohort of 26 patients with different chromosomal alterations involving the *KMT2A* gene has revealed that patients with the t(4;11) translocation, which corresponds to the worst outcome subtype of BCP-ALL, have much more highly hypomethylated enhancer sites than normal BCPs and other KMT2A-rearranged subtypes [57]. The reported hypomethylation sites involve several genes that are differentially expressed in t(4;11) BCP-ALL and thereby contribute to leukemogenesis [57]. This set of genes includes *HDAC4* and *MSI2*, which are correlated with adverse outcomes in *KMT2A*-rearranged BCP-ALL [120,121] and, more importantly, *DOT1L*, whose function in triggering progression of leukemogenic cells in this context is described in Section 2.2.

### 2.4. Histone acetylation Alterations

The acetylation of histone lysine residues is one of the most widely documented histone modifications. Alterations in histone acetylation occur in several cancers, including childhood BCP-ALL [60,61,62]. Global acetylation profile studies have reported a global loss of H4 acetylation associated with poor outcome [63], while a deficit in H3 acetylation is correlated with prednisolone resistance in xenograft murine models, driven by the epigenetic silencing of the *BIM* gene [64].

The regulation of histone lysine acetylation is controlled by a variety of enzymes, the most important of which are the lysine acetyltransferases (KATs) and histone deacetylases (HDACs) [60]. The acetyltransferase CREBBP, whose expression is altered in a variety of cancers, is one of these enzymes [60,62,65]. In the context of BCP-ALL, CREBBP is commonly mutated or deleted, and these alterations usually affect the histone acetyltransferase (HAT) domain. Since this CREBBP function is regularly impaired in relapsed patients but is rare in non-relapsing cases, it is believed to induce chemotherapy resistance [122]. The mutations impair acetylation of histones and transcriptional activation of CREBBP targets, including genes that are important for conferring glucocorticoid sensitivity on BCP-ALL cells, like *RGS16* or *DUSP10* [113,114]. CREBBP mutations are also particularly prevalent in cases of BCP-ALL with hyperdiploidy [123].

Likewise, the CREBBP homolog *EP300* is involved in a gene fusion (EP300-ZNF384). This is proving to be a novel genomic alteration in BCP-ALL, with an incidence around 2% in infants, and greater than 5% in adolescents and young adults [66,124]. This fusion protein not only “reads” chromatin modifications, but also recruits other proteins to the chromatin, for which reason, inhibiting its bromodomain regions, is considered a potential therapeutic strategy [125]. A transcriptomic analysis of samples harboring this alteration shows an aberrant activation of the JAK/STAT pathway, driving leukemia progression [66]. Under normal conditions, the KLF4 transcription factor is acetylated by EP300 and mediates TP53 action by blocking PI3K signaling [126]. When the ZNF384-EP300 fusion protein is expressed, EP300 loses its activity, provoking KLF4 and TP53 dysregulation that, combined with JAK/STAT activation, causes the uncontrolled progression of overt BCP-ALL [66].

**Figure 2:**
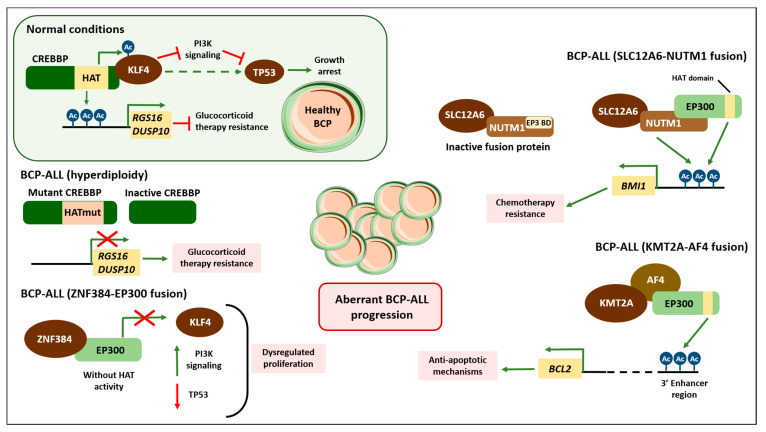
Alterations of histone acetylases drive aberrant BCP-ALL progression. Histone acetylase CREBBP and its homolog EP300 are involved in driving progression of distinct subtypes of BCP-ALL, which displayed the following alterations: *ZNF384*-*EP300*, *SLC12A6*-*NUTM1* and *KMT2A*-*AF4* fusions and hyperdiploid BCP-ALL. Inset displaying the role of CREBBP under healthy conditions is colored green. Green arrows indicate activation; red lines and crosses indicate repression. Red squares indicate processes that induce a poorer prognosis. Blue marks labeled “Ac” indicate global histone acetylation, while purple “Ac” marks indicate acetylation of H3K27 residue in the enhancer region of BCL2 (in the context of the *KMT2A*-*AF4* gene rearrangement). EP3 BD refers to EP300 Binding Domain.

A novel recurrent genetic alteration has recently been described in BCP-ALL that consists of an in-frame *SLC12A6-NUTM1* fusion [67]. The resulting transcript contains exons 3–8 of the *NUTM1* gene and is predicted to encode a chimeric protein, with a binding domain for EP300 [68]. When EP300 is recruited, the SLC2A6-NUTM1 protein acquires transcriptional activity and thereby induces key oncogenic drivers like BMI1, a marker of chemotherapy resistance [67]. A summary of how CREBBP and EP300 mutations induce aberrant activation of BCP-ALL is depicted in Figure 2.

The overexpression of some enzymes of the HDAC family (HDAC2, −3, −4, −6, −7, −8, −9, −11) has been linked to an unfavorable prognosis and an impaired response to conventional glucocorticoid treatment in two studies [120,127]. However, these studies included both B- and T-cell ALL and their associated transcriptional profiles differed profoundly. In fact, the importance of HDAC proteins has been less thoroughly studied even than histone acetyltransferases, and no direct causal association with HDACs has been irrefutably identified [128]. Accordingly, a recent study in our laboratory that looked at BCP-ALL patients with the t (4;11) translocation demonstrated that this aggressive leukemic subtype has almost negligible levels of HDAC7 expression, unlike other subtypes with better outcomes [129]. Therefore, it is tempting to speculate that targeted therapy would benefit from the selective inhibition of specific HDAC proteins, rather than from a complete family blockade.

However, several studies of the use of pan-HDAC inhibitors such as LBH589 (panobinostat) and TSA (trichostatin A) have yielded promising results in terms of synergistic effects with common chemotherapeutic agents [130,131,132]. Both drugs impose global H3 acetylation, as well as specific acetylation at H3K9 residues in BCP-ALL cells, which compensate the repressive effect of promoter methylation on some tumor suppressor genes, such as the protocadherin *PCDH17* [131,132]. Additionally, an unexpected effect on another epigenetic modification, in which H2BK120 ubiquitination was lost upon LBH589 treatment, was found only in *KMT2A*-rearranged cells [131]. This modification is discussed further in Section 2.5.

KMT2A rearrangements affect not only the methylation profile of target genes, but also histone acetylation, as exemplified by the regulation of anti-apoptotic genes such as *RUNX1*, *MCL1,* and *BCL2*. The cooperation between KMT2A-AF4 and DOT1L induces their expression through the increased methylation of the H3K79 residue [133]. However, the KMT2A-AF4 fusion protein can also induce BCL2 expression by recruiting EP300 acetyltransferase to an enhancer region located in a 3′ region of the *BCL2* gene, which enriches the H3K27ac activating mark in this region [56]. The greater degree of acetylation in these regions is facilitated by the hypomethylation displayed in CGIs located within enhancers [57]. KMT2A-AF4 also associates with other acetyltransferases, such as MOF, which acts on H3K9 and H4K16, imposing active marks such as H4K16ac on promoter regions [59].

In addition to *AF4*, *KMT2A* can translocate to other partners, like *ENL*, that also induce typical BCP-ALL markers, such as *HOXA9* and *MEIS1* [134]. ENL is a potential target in *KMT2A-AF4* leukemia, since it is needed to maintain aberrant in vivo and in vitro proliferation, mediated by its YEATS chromatin-reader domain [135]. When ENL is pharmacologically degraded, rapid cell cycle arrest is induced, together with a clear downregulation of *HOXA10*, *MYC*, *MYB,* and *MEIS1* expression. Instead, myeloid markers like *CD11B* are upregulated. ChIP-seq data show that ENL binds to promoter regions of these targets, enriched in H3K9ac, H3K18ac and H3K27ac through the YEATS domain [135], which have been reported as being gene activation marks [56,58,59]. These data indicate that *KMT2A*-rearranged BCP-ALL can also progress through alternative mechanisms in a DOT1L-independent fashion.

### 2.5. Histone Ubiquitination Alterations

The histone H2A mono-ubiquitylation mark confers low transcriptional activity on genes. Conversely, H2B ubiquitination at Lys120 (H2BK120ub) induces transcriptional elongation and facilitates H3 methylation [69]. The methylation of H3K4 and H3K79 residues, mediated by KMT2A and DOT1L enzymes, respectively, take place through an H2B-ubiquitin-dependent mechanism [70,71]. In the context of *KMT2A*-rearranged BCP-ALL, mechanistic studies have sought to define how DOT1L is aberrantly activated and leads to an excess of H3K79 methylation [69,82]. The disulfide-directed methodology developed by Chatterjee et al. [69] revealed that the stimulatory effect not only occurs upon H2B ubiquitination in Lys120 but can also be mirrored by ubiquitination at other positions in the K120 surrounding area. For instance, H2BK125ub, whose specific function in human cells is not known, exhibits similar activity to that of the native K120 site [69].

The localization pattern of the H2Bub chromatin mark resembles that of H3K79me2. Importantly, the knockdown of KMT2A-AF9 fusion protein in t(9;11)-rearranged ALL cells triggers a concomitant downregulation of both H3K79me2 and H2Bub marks, indicating that these two events, which cooperate in RNA polymerase II transcription elongation [81], are somehow interconnected [136]. Additionally, the presence of H2Bub mark is required to maintain DOT1L activity and, therefore, to keep high levels of H3K79me2 in KMT2A-fusion proteins targets [136].

The incorporation of the H2BK120ub mark depends on the BRE1 protein complex, which includes the protein-protein adaptor WAC and ubiquitin ligase RNF20, which is the major E3 ligase that catalyzes ubiquitination of H2B in mammalian cells [137]. RNF20 has been defined as an essential regulator of chromatin accessibility, required for leukemogenesis mediated by KMT2A-fusion proteins [136]. The pan-HDAC inhibitor LBH589 provokes RNF20 downregulation in patients with *KMT2A* rearrangement, inducing not only acetylation loss, but also a decrease in the levels of H2BK120ub mark [131].

## 3. Concluding Remarks

As described throughout this review, strict control of the epigenetic mechanisms that drive B lymphocyte differentiation can be easily disrupted at several points, giving rise to infant BCP-ALL. In fact, the initial hit occurring during embryogenesis commonly involves chromosomal rearrangements affecting genes with methyltransferase activity (such as *KMT2A*), resulting in increased catalytic activity that is exacerbated by interaction with other cofactors. Remarkably, methylation, acetylation or ubiquitination of several histone residues (H2BK120, H3K4, H3K9, H3K27, H3K36, H3K79, H4K16, and H4R3) can trigger a variety of processes related to leukemogenic cell capacity, either inducing or repressing it. In some specific residues, such as in the case of H3K27, acetylation and methylation determine the accessibility of transcription factors to chromatin. However, depending on the genomic region involved, the epigenetic modification of histone residues has distinct outputs in terms of cell viability, aberrant proliferation and/or resistance to chemotherapy. In consequence, obtaining a full epigenomic profile of BCP-ALL infants would promote the development of novel therapies focused on reverting key epigenetic alterations that drive this malignancy. As an example, an integrative epigenomic analysis performed in adult B-ALL provided the basis for a clinical trial with BCL6 inhibitors in specific cases of B-ALL with a concrete epigenetic alteration [138].

Apart from providing a better understanding of the molecular effects beyond epigenetic gene regulation, we provide further insight into the clinical relevance of epigenetic modifications. For instance, the DNA methylation signature can be used to assign BCP-ALL cases to a specific subtype and to predict disease outcome, while loss-of-function mutations in methyltransferases (such as NSD2) or KATS (like CREBBP or EP300) may be used to predict prognosis and response to chemotherapy. In this sense, the epigenetic alterations reported here constitute a potential entry point for therapeutic approaches for BCP-ALL. In fact, compounds blocking DOT1L activity or impairing binding between KMT2A-AF4 aberrant protein and its cofactor Menin are already undergoing clinical trials for the treatment of hematological diseases [85,90], with the promise of a novel treatment for BCP-ALL in the near future. Therefore, we believe that further research on epigenetic processes will give rise to effective therapies that, alone or in combination with current therapies, will improve the prognosis of infant BCP-ALL.

## Figures and Tables

**Table 1:** Common epigenetic histone modifications in B-cell precursor acute lymphoblastic leukemia (BCP-ALL).

Histone Mark	Regulators	Effect on Chromatin Conformation	Alteration of Gene Expression and Biological Processes	References
*Histone methylation*				
H3K4me3	KMT2A chromosomal rearrangements Cofactors: WDR5, RbBP5, ASHL2, DPY-30, Menin PAF1 and E2/E3 complexes	Transcriptionally active chromatin, that can turn into repressive state, depending on other marks	Regulation of proper B lymphocyte development Oncogenic drivers, such as *FLT3* It can activate *IKZF1* gene	[24,25,26,27,28,29,30,31,32,33,34]
H3K79me3	Members of super elongation complex (SEC), such as AF4, AF9 or ENL DOT1L, NSD1, CARM1 PAF1 and E2/E3 complexes	Increased chromatin accessibility, allows binding of transcription factors to promoter regions	Negative outcome markers: *CPEB2*, *MBNL1*, *MCL1*, *RUNX1*, *RUNX2*, *ZEB2*	[29,35,36,37,38,39,40,41]
H3K9me3	IKZF1, SIRT1	Repressive chromatin state	Repression of genes involved in cell cycle progression (*CDC2*, *CDC7*)	[40,42,43,44]
H3K36me2/3	Catalyzed by NSD2	Present at bodies of transcriptionally active genes, impairing aberrant transcriptional initiation	This mark impairs aberrant leukemogenic activity. NSD2 mutation triggers cell proliferation	[45,46,47,48,49]
H3K27me3	EZH2, IKZF1, NuRD repressive complex (including HDAC1, HDAC2 and MI-2)	Close chromatin conformation	Tumor suppressor function, preventing cell cycle progression	[43,44,45,50,51]
H4R3sme2	PRMT5	Closed chromatin conformation at promoter regions	Repression of genes involved in proper B cell differentiation and apoptosis (like *CLC* and *CTSB*)	[52,53,54]
*Histone acetylation*				
H3K27ac	EZH2 loss-of-function, EP300, KMT2A-AF4 fusion protein	Increased chromatin accessibility in enhancer regions, recruiting CTCF, GR and PU.1 factors	Induction of *BIM* target gene, marker of glucocorticoid sensitivity. Activation of *BCL2* antiapoptotic gene and oncogene *FLT3*	[33,45,55,56]
H3K9ac and H4K16ac	KMT2A-AF4 fusion protein, in association with MOF	Active marks on promoter regions, allowed by hypomethylation pattern at enhancer regions	Activation of *BCL2* antiapoptotic gene	[56,57,58,59]
Global H3 and H4 acetylation loss	KATs (such as CREBBP), HDACs and fusion proteins derived from genetic alterations (SLC12A6-NUTM1 or ZNF384-EP300)	Chromatin silencing through the imposition of a repressive state	Poor outcome, associated to loss of H4 acetylation Blockade of *BIM* Prednisolone treatment resistance in murine models with deficit of H3 acetylation (impairing *Rgs16* or *Dusp10* induction)	[58,60,61,62,63,64,65,66,67,68]
*Histone ubiquitination*				
H2BK120ub	BRE1 protein complex (including WAC and RNF20)	Promotes transcriptional elongation by facilitating H3 methylation	Presence of H2BK120ub residues mediates KMT2A and DOT1L activity, maintaining BCP-ALL progression	[69,70,71]

## Data Availability

Not applicable.
